# Transcultural adaptation of the Thirst Distress Scale (TDS) into Brazilian Portuguese and an analysis of the psychometric properties of the scale for patients on hemodialysis

**DOI:** 10.1590/2175-8239-JBN-2019-0151

**Published:** 2020-03-13

**Authors:** Clara Sandra de Araujo Sugizaki, Clarice Carneiro Braga, Ana Tereza Vaz de Souza Freitas, Maria do Rosário Gondim Peixoto

**Affiliations:** 1 Universidade Federal de Goiás, Faculdade de Nutrição, Programa de Pós-Graduação em Nutrição e Saúde, Goiânia, GO, Brasil.

**Keywords:** Renal Dialysis, Thirst, Validation Studies, Translations, Surveys and Questionnaires, Diálise Renal, Sede, Estudos de Validação, Traduções, Inquéritos e Questionários

## Abstract

**Objective:**

To produce a transcultural adaptation of the Thirst Distress Scale (TDS) into Brazilian Portuguese and analyze the scale’s psychometric properties for patients on hemodialysis (HD).

**Methods:**

The original scale was translated, back translated, and discussed with psychometric assessment experts. The final version was tested with 126 patients on HD and retested with 70 individuals from the original patient population. Cronbach’s alpha was used to measure the scale’s internal consistency. Reliability of thirst intensity evaluated via the visual analogue scale (VAS) was tested with Kappa statistic and the Bland-Altman plot. Reproducibility was assessed based on the intraclass correlation coefficient (ICC).

**Results:**

The wording of three items and the verb tenses of six had to be adjusted in the final version of the Brazilian Portuguese TDS. Comprehension of the scale by patients on HD was good, the scale’s internal consistency was satisfactory (0.84; p<0.001), agreement with a visual analogue scale (VAS) was moderate (kappa=0.44; p<0.001), and reproducibility neared perfection (ICC=0.87; p<0.001).

**Conclusion:**

Our results showed that the Brazilian Portuguese version of the scale might be used reliably. The Brazilian Portuguese version of the TDS is a practical, affordable, accessible and well-accepted tool that has a lot to offer for the management of patients with HD.

## Resumo

**Objetivo:**

Realizar a adaptação transcultural da escala Thirst Distress Scale (TDS) para o português brasileiro e estudar suas propriedades psicométricas em pacientes em hemodiálise (HD).

**Métodos:**

Foram realizadas traduções, retrotraduções, discussão com especialistas e avaliação psicométrica, com aplicação da versão final em 126 pacientes em HD e reteste em 70 pacientes da amostra inicial. A consistência interna do instrumento foi obtida pelo alfa de Cronbach. Para analisar a concordância com a intensidade de sede, avaliada pela Escala Visual Analógica (EVA), foi utilizado o teste Kappa e a estratégia gráfica de Bland-Altman. Para avaliar a reprodutibilidade, foi realizado teste de correlação intraclasse (CCI).

**Resultados:**

Para obtenção da versão final da escala TDS em português brasileiro, intitulada TDS-BR, foi necessária adaptação de vocabulário em três itens e mudança de tempo verbal em seis itens. Houve boa compreensão da escala pelos pacientes em HD, consistência interna satisfatória (0,84, p<0,001), concordância moderada com a Escala Visual Analógica (EVA) (kappa=0,44; p<0,001) e reprodutibilidade quase perfeita (CCI=0,87; p<0,001).

**Conclusão:**

Os resultados obtidos indicam a aplicabilidade e confiabilidade do instrumento na língua portuguesa (Brasil). A ferramenta, por ser de fácil compreensão e baixo custo, além de ter boa aceitação, pode ser um instrumento relevante no manejo da sede de pacientes em HD.

## Introduction

Thirst is a sensation that cannot be ignored. It is a threshold symptom indicative of fluid imbalances stemmed from dehydration or increased plasma solute concentration.^1^ In theory, a one-percent increase in osmolality may trigger sensations of thirst.^2^ In some noncommunicable diseases (NCDs) - diabetes mellitus,^3^ heart failure,^4^ some types of cancer,^5^ and end-stage renal disease (ESRD)^6^ - thirst is a recurring symptom.

Chronic kidney disease (CKD) is a global public health problem. An estimated six million individuals suffer from renal impairment in Brazil,^7^ and more than 133,000 have ESRD.^8^ The choice of renal replacement therapy (RRT) for more than 90% of the individuals with ESRD is hemodialysis (HD).^8^ Fluid restriction ranks high among the difficulties reported by individuals on HD due to the distress inherent to feeling thirsty.^9^^,^ 10 Distress has been defined as the suffering and discomfort caused by a symptom.^6^ Distress caused by thirst is a common finding in individuals on HD, since patients are prescribed fluid restriction^11^ and present with increased plasma osmolality secondary to sodium retention during the period between dialysis sessions, which incessantly stimulates a sensation of thirst.^12^ Another possible trigger is the combination of hypotension and hypovolemia immediately after HD.^13^The quality of life of patients on HD is significantly affected as they are in distress for feeling constantly thirsty and not being allowed to drink fluids.^9^^,^ 10

Although thirst has a subjective facet, scales have been used to diagnose and assess the thirst of patients on HD in terms of intensity (visual analogue scale - VAS),^14^ frequency (Dialysis Thirst Inventory - DTI),^15^ and distress (Thirst Distress Scale-TDS).^16^^,^ 17 The TDS is the only scale adapted and validated in other countries, including Turkey,^18^ Japan,^19^ Sweden,^19^ the Netherlands,^19^ and Italy.^20^ The TDS is a 6-item scale. Each item is rated based on a 5-point Likert scale (0 = strongly disagree; 5 = strongly agree). Results are given in a score ranging from 6 to 30 points, in which respondents are rated as having mild (score < 10), moderate (scores between 10 and 18), or severe thirst (score > 18). In the original publication patients were interviewed, but the questions in the scale may also be answered by respondents alone, since it is a reliable, easy-to-use tool with adequate internal consistency.^16^

There is no scale currently validated for use in Brazilian Portuguese to rate distress resulting from thirst. The need for a validated scale lies in the difficulty inherent to assessing thirst in clinical settings^21^ and in the relevance of thirst for patients on HD and its ties to fluid restriction and interdialytic weight gain.^6^^,^ 15 This paper presents a transcultural adaptation of the TDS scale into Brazilian Portuguese and offers an analysis of the scale’s psychometric properties for patients on HD.

## Methods

This cross-sectional study was designed to assess the Thirst Distress Scale in Brazilian Portuguese for semantic equivalence and validate it. The study included 126 patients, a sample size characteristically seen in validation studies.^23^^-^ 26 Data collection took place in the morning, afternoon, and evening shifts during HD sessions. The questionnaire included identification information and probed into etiology of CKD, time on HD, KT/V (a marker of quality of dialysis), comorbidities, body mass index (BMI), interdialytic weight gain (an indicator of fluid status), biochemical tests (transferrin, hemoglobin, ferritin, albumin, creatinine, pre-dialysis blood urea nitrogen, post-dialysis blood urea nitrogen). Patients were analyzed for drug-induced dry mouth and prescription of loop diuretics (furosemide), aldosterone antagonists (spironolactone), ACE inhibitors (captopril, cilazapril, enalapril, fosinopril, lisinopril, ramipril, perindopril), angiotensin II receptor blockers (losartan, candesartan, eprosartan, irbesartan, telmisartan, valsartan), and vasopressin receptor antagonists (tolvaptan).^4^

Our study enrolled patients from two private HD clinics in Goiânia, Brazil. Clinically stable male and female patients on three HD sessions a week aged 18-79 years treated for at least three months were included. Patients on HD for less than three months and individuals diagnosed with gastrointestinal tract disorders, cognitive or eyesight impairment were excluded.

The author of the original study consented to the changes introduced in our adaptation.^16^. The cross-cultural adaptation procedures were carried out based on the recommendations set out in the Guidelines For The Process Of Cross-Cultural Adaptation Of Self-Report Measures. The process included translations, back translations, meetings with expert committees, and pretesting before a final scale in Brazilian Portuguese was obtained. This study was divided into two stages: a first stage in which a Brazilian Portuguese version of the TDS was proposed; and a second stage in which the scale’s psychometric validity and reliability properties were assessed.

### Step I: transcultural adaptation of the TDS into Brazilian Portuguese

**Figure 1:**
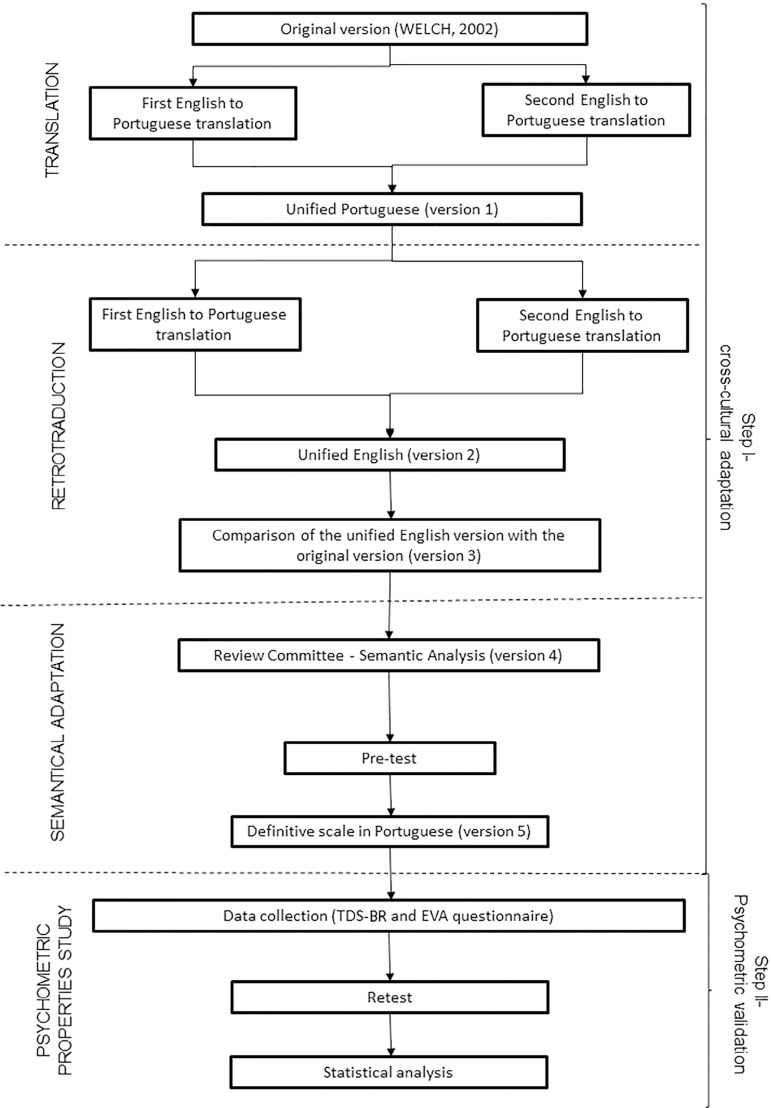
Study design

The TDS^16^ questionnaire was translated from English into Portuguese by two translators/nutritionists who had not seen the scale before translating it. The two were native speakers of Portuguese and spoke English fluently. The two translations were compared and merged into one single version by the two translators (version 1). Two native speakers of English working in healthcare who had not seen the original questionnaire back translated the merged version of the scale into English. The two back-translated documents were merged (version 2). An expert bilingual translator worked with the original scale in English and the back-translated version in English to produce a semantically equivalent version of the document (version 3). This technical review allows the verification of the equivalence between the original scale in English and the back-translated version of it so as to ensure that denotative meaning has been conveyed from one into the other (Figure 1).

A panel with 12 experts - physicians, nutritionists, and nurses involved in HD - reviewed the document and introduced semantic adaptations to reflect local colloquialisms and cultural preferences. Version 4 of the document captured the general connotative meaning of each item. As in the original scale, the six sentences were tied to a 5-point Likert scale (0 = strongly disagree; 5 = strongly agree).^16^

A pretest run was carried out to verify each item for respondent comprehension and standardize the approach adopted by interviewers. Samples assembled for this purpose usually contain a minimum of 30 individuals.^22^ The pretest run included 55 randomly picked patients on HD (< 20 years) involved solely on the pretest run. Only then was the fifth and final version of the scale in Portuguese produced (end of transcultural adaptation) and analyzed for psychometric properties.

### Step II: analysis of the psychometric properties of the TDS-BR

The patients were assessed for thirst distress two hours into their HD sessions. They were asked questions about the distress they felt for feeling thirsty since their more recent HD session. The patients were interviewed by one of four trained interviewers.^16^^,^ 18 ^-^ 20

The lack of a gold standard against which to validate a scale has led other authors^6^^,^ 18 ^-^ 20 ^,^ 22 to make comparisons against a VAS, a validated instrument to measure the intensity of pain^14^ broadly used in the assessment of thirst.^6^^,^ 16 ^,^ 18 ^-^ 20 ^,^ 22 This procedure is based on the Theory of Symptom Management,^25^ in which converging validity is assigned when theoretically related dimensions present associations in reality. The validity of the framework stands on the fact that more intense thirst is expected to cause greater distress.

The VAS was printed in a 10-cm paper slip showing a gradation of colors (from green to red) to illustrate gradual intensity increases.^14^ Patients were advised to mark the option that best characterized their thirst since the last HD session with an ‘X,’ in a scale in which zero meant no thirst and 10 maximum thirst.^14^ Scores were assigned based on the distance in centimeters between point zero and the point marked by the patients to characterize thirst intensity.^16^^,^ 18 ^-^ 20

Within a week, 70 participants (55% of the original population) were retested and asked to answer the questions on thirst from the questionnaire (VAS and TDS-BR); they followed the same procedure adopted the first time and the scale was assessed for reproducibility (Figure 1).

The Ethics Committee with the Federal University of Goiás (UFG) approved the study protocol and assigned it certificate no. 54523116500005083. Included patients gave written consent before joining the study.

### Statistical analysis

Data points were entered into Microsoft Excel for Windows. Data analysis was performed on software package Stata version 12.0. Continuous variables were tested for normality with the Shapiro-Wilk test. Absolute and relative frequencies were calculated for categorical variables; mean values and standard errors were calculated for continuous variables following a normal distribution; and medians and interquartile ranges (p25-p75) were computed for non-parametric variables. The differences between variable mean/median values were calculated using Student’s t-test, ANOVA, the Mann-Whitney U test, or the Kruskal-Wallis test depending on data distribution.

The validity of the TDS-BR was tested for internal consistency (Cronbach’s alpha), correlation coefficient (Pearson’s or Spearman’s), agreement with thirst intensity data interpreted from the VAS (Kappa statistic and Bland-Altman), and reproducibility (interclass correlation coefficient). Statistical significant was attributed to events with a *p*<0.05.

## Results

Transcultural adaptation and analysis of psychometric properties supported the consolidation of an agreed version of the scale in Brazilian Portuguese referred to as the Thirst Distress Scale - Brazilian Portuguese (TDS-BR).

After reading the Brazilian Portuguese version of the scale, the panel of experts unanimously described the first three items as indistinguishable. In order to resolve the issue, the word “muito” (item 3, the proposed translation for *very*) was changed to “bastante” (another adverb of degree). The change allowed a clearer perception of increasing intensity from the first to the third item. Thus, item 3 expressed greater discomfort than item 2. In the Brazilian Portuguese version, clause *feels like cotton* was translated as “ficou seca como algodão” (it felt as dry as cotton), since the allusion to the dryness of cotton is not commonly used to refer to dry mouth in Brazil. The word “líquido” (liquid) was added to item 6 to further clarify the meaning of the question (Table 1).

**Table 1:** The original version of the Thirst Distress Scale (TDS) versus the Brazilian Portuguese version of the TDS (TDS-BR)

Original version	Brazilian Portuguese version	Version produced after input from the panel of experts	Final version
1) *My thirst causes me discomfort.*	Minha sede me causa desconforto.	Minha sede me causa desconforto.	Minha sede me **causou** desconforto.
2) *My thirst bothers me a lot.*	Minha sede me incomoda muito.	Minha sede me incomoda muito.	Minha sede me **incomodou** muito.
3) *I am very uncomfortable when I am thirsty.*	Eu fico muito desconfortável quando estou com sede.	Eu me sinto **bastante** desconfortável quando eu estou com sede.	Eu me **senti** bastante desconfortável quando eu **estive** com sede.
4) *My mouth feels like cotton when I am thirsty.*	Minha boca parece algodão quando estou com sede.	Minha boca **fica seca como** algodão quando eu estou com sede.	Minha boca **ficou** seca como algodão quando eu **estive** com sede.
5) *My saliva is very thick when I am thirsty.*	Minha saliva fica muito grossa quando estou com sede.	Minha saliva fica muito grossa quando eu estou com sede.	Minha saliva **ficou** muito grossa quando eu **estive** com sede.
6) *When I drink less, my thirst gets worse.*	Quando eu bebo menos, minha sede fica pior.	Quando eu bebo menos **líquido**, minha sede fica pior.	Quando eu **bebi** menos líquido, minha sede **ficou** pior.

The patients participating in the pretest of the adapted scale were not involved in other steps of the process. Some 80% of the patients had trouble understanding that the thirst alluded to in the questionnaire referred to the thirst from their most recent HD session. A second meeting with the panel of experts resulted in the reformulation of the translated questions. Verbs were switched from the present - as in the English version of the scale - to the past tense.

In regard to sociodemographic and clinical variables, 61.11% of the patients were males; their mean age was 53 years (±16.59) and the median time for which they had been on HD was 44.5 months (20-89). The most prevalent comorbidity was systemic hypertension (80.95%). Based on the BMI, the patients were in good nutritional status (Table 2).

**Table 2:** Characterization of the patient population involved in Step II of the study

Variables*	Total
**Sex**	
Male	77 (61.11%)
Female	49 (38.89%)
Age (years)	53.00 (± 16.59)
Time on hemodialysis (months)	44.50 (20.00-89.00)
KT/V **	1.39 (± 0.23)
**Etiology of chronic kidney disease**	
Hypertensive nephrosclerosis	40 (32.74%)
Glomerulonephritis	9 (7.14%)
Diabetic nephropathy	9 (7.14%)
Adult polycystic kidney disease	11 (8.75%)
**Comorbidities**	
*Diabetes mellitus* II	15 (11.90%)
Systemic hypertension	102 (80.95%)
Body mass index (kg/m^2^)	24.85 (21.70-28.95)
Interdialytic weight gain (kg)	1.80 (± 0.90)
**Biochemical tests**	
Hemoglobin (g/dL)	11.50 (10.40-13.00)
Transferrin (%)	33.00 (25.30-42.90)
Ferritin (ng/mL)	239.50 (25.00-354.40)
Albumin (g/dL)	3.70 (± 0.50)
Creatinine (mg/dL)	11.20 (± 3.10)
Pre-dialysis blood urea nitrogen (mg/dL)	126.00 (± 32.50)
Post-dialysis blood urea nitrogen (mg/dL)	29.50 (22.00-42.50)
Use of drugs that may cause dry mouth	102 (80.95%)

* Values presented as numbers (proportions), mean value ± standard error or median and interquartile interval.

** KT/V: pre-dialysis blood urea nitrogen/post-dialysis blood urea nitrogen

Internal consistency was rated as satisfactory based on Cronbach’s alpha (0.84; p<0.001). In order to assess the impact of each item in the internal consistency of the scale, Cronbach’s alpha was calculated after removing each question at a time (Table 3). A comparison of each estimated punctual alpha revealed that the removal of items did not significantly increase the punctual estimation of alpha. Therefore, all items were kept. The removal of item 6, the weakest in the scale, caused the highest increase in Cronbach’s alpha (0.86). The correlations between TDS-BR items were within the expected boundaries (0.3 and 0.85) (Table 4).

**Table 3:** Internal consistency per item in the TDS-BR (Thirst Distress Scale – Brazilian Portuguese)

Final version of the scale	$\overset{´}{\chi }$	SE	Total correlation	Cronbach’s alpha for each item deleted
1) Minha sede me causou desconforto.	2.86	1.59	0.77	0.80
2) Minha sede me incomodou muito.	2.88	1.60	0.84	0.79
3) Eu me senti bastante desconfortável quando eu estive com sede.	3.00	1.65	0.86	0.78
4) Minha boca ficou seca como algodão quando eu estive com sede.	3.50	1.54	0.74	0.82
5) Minha saliva ficou muito seca quando eu estive com sede.	2.76	1.75	0.68	0.83
6) Quando eu bebi menos líquido, minha sede ficou pior.	2.89	1.54	0.56	0.86

$\overset{´}{\chi }$ mean score for each item;

SE: standard error.

**Table 4:** Correlation matrix for items in the TDS-BR (Thirst Distress Scale – Brazilian Portuguese)

	Item 1	Item 2	Item 3	Item 4	Item 5	Item 6
Item 1	1.000					
Item 2	0.783	1.000				
Item 3	0.627	0.754	1.000			
Item 4	0.456	0.482	0.594	1.000		
Item 5	0.339	0.408	0.529	0.534	1.000	
Item 6	0.317	0.348	0.353	0.268	0.229	1.000
a	100%	100%	100%	80%	80%	60%
b	0.504	0.555	0.571	0.467	0.408	0.303

a Proportion or times for which the two-by-two correlations ranged between 0.30 and 0.85

b Mean correlation of each item with other items in the scale

Statistical test: Pearson's correlation coefficient

Thirst intensity assessed via the VAS^23^ (Table 5) found that most patients had moderate thirst. The mean score patients attained in the TDS-BR also indicated moderate thirst (17.87; ±7.87). When the VAS scores were matched against the TDS-BR scores, a clear association emerged between severe thirst and greater distress (p<0.001).

**Table 5:** Distribution of thirst intensity and comparison against the TDS-BR (Thirst Distress Scale – Brazilian Portuguese)

Thirst intensity (EVA)	N (%)	TDS-BR	
		Mean*	p
Mild	24.60	12.13(±5.57)	< 0.001
Moderate	55.56	17.54(±6.11)	
Severe	19.84	23.72(±4.81)	

* Statistical differences between the three groups (mild, moderate, and severe thirst) by the Kruskal-Wallis test, p < 0.05

**Figure 2:**
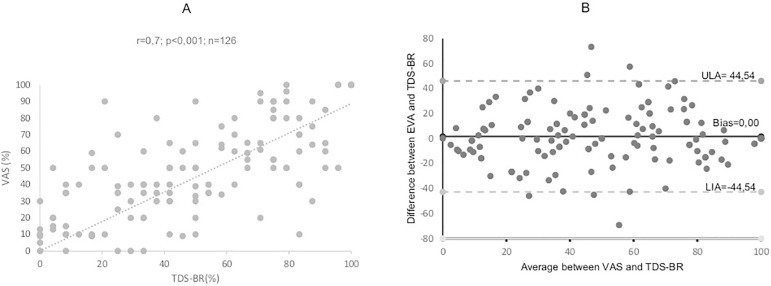
(A) Agreement scatter between VAS (%) and TDS-BR (%). (B) Bland-Altman plot used to assess agreement between the VAS (%) and TDS-BR (%). Bias = mean difference. ULA = upper limit of agreement. LLA = lower limit of agreement

A moderate correlation was found when the scores in the two scales (Figure 2A) were analyzed (r = 0.70; p< 0.001). The Bland-Altman plot (Figure 2B) showed a homogeneous distribution with a non-significant estimated bias of zero. However, the limits of agreement were rather far apart (±44.54). Kappa was calculated to estimate the agreement between the categories in the VAS and the TDS-BR. A Kappa of 0.44 (p < 0,001) indicated moderate agreement.

The test and retest scores were not significantly different. The reproducibility of the Brazilian Portuguese version of the TDS neared perfection, with an intraclass correlation coefficient of 0.87 (p<0.001) (Table 6).

**Table 6:** Comparison between TDS-BR (Thirst Distress Scale – Brazilian Portuguese) test and retest and correlation (N = 70)

	Test	Retest	t^a^	p	ICC (95% IC)
TDS-BR	18.28 (±7.22)	18.01 (±7.55)	0.84	0.201	0.87 (0.77- 0.97)*

ICC = intraclass correlation coefficient;

CI = confidence interval;

paired t-test

* p < 0.001

## Discussion

The internal consistency of the transcultural and psychometric adaptation of the scale had satisfactory internal consistency, moderate agreement with the VAS, and nearly perfect reproducibility. Our results revealed that the TDS-BR might be reliably used to diagnose and assess distress resulting from thirst in patients on HD.

The replaced and included terms drew the TDS-BR closer to the semantics intended in the original scale.^16^ Other transcultural adaptation attempts have also included changes to the original instruments. In the scale adapted for patients with heart failure, “algodão” (cotton) was replaced with “lixa” (sandpaper),^19^ a more adequate term in Portuguese to express a sensation of roughness rather than dryness.^24^ In the Turkish adaptation, the term “cotton” was suppressed and the item read “my mouth gets really dry when I am thirsty.”^18^ Item 5 remained unchanged in our and other adaptations.^18^^,^ 19 We added the word “líquido” (liquid) in item 6 and the word “water” was added in a multicenter study.^22^ The verb tenses in the other adaptations remained unchanged in the present tense. The author of the original study was informed of the changes introduced in our adaptation.

The elevated level of internal consistency seen in our adaptation was also observed in other validations of the instrument, with values ranging between 0.78^16^ and 0.81.^18^ Item 4 had the highest mean score, indicating that having severe dry mouth led to significant distress. This finding was consistent with the mean values reported in the literature.^15^^,^ 16 ^,^ 18 ^,^ 19 The weakness found in item 6 also appeared in the English,^16^ Turkish,^18^ Japanese,^19^ Swedish,^19^ and Dutch^19^ versions of the scale.

The correlation between the three items was more significant than the correlations between other items. The last three items were moderately correlated. Therefore, none of the items had to be removed. Other TDS validation efforts kept the three items. Kara (2013)^18^ did not make changes and Waldréus et al. (2017)^19^ changed item 2 to “my thirst bothers me every day” and kept the other items unchanged.

The mean TDS-BR scores indicated that patients on HD had moderate thirst. The author of the original study reported similar mean scores (17.1; ±4.2).^14^ The mean scores in the Turkish and Italian versions of the TDS indicated the occurrence of severe thirst (20.32; ±4.23^31^ and 21.4 ±4.2,^57^ respectively).

TDS-BR scores were positively correlated with VAS scores. Although the two scales were in agreement, they cannot replace one another. A Bland-Altman plot showing a non-significant difference of zero indicates good agreement between the two. Significantly far apart upper and lower limits indicate “error” and the possibility of responses oscillating between scales.^26^^-^ 29 Reproducibility in the TDS-BR neared perfection, as also described by other authors.^16^^,^ 18 ^,^ 19

Other instruments have been used to assess thirst in different dimensions.^15^^,^ 30behaviors, and attitudes of hemodialysis (HD However, there is no gold standard method to compare these instruments against. The TDS was chosen for its ease-of-use and effective transcultural adaptation into different languages.

The transcultural adaptation reflected in the TDS-BR was successfully tested with a group of patients on HD from Goiânia, Brazil. Although the sentences in the scale are enunciated in the first person singular - suggesting the questionnaire might not require an interviewer, the questions were asked to the patients with the aid of an interviewer as in the original study^16^ and in other adaptations of the scale.^6^^,^ 18 ^-^ 20 ^,^ 22 Future studies should look into response agreement between different questionnaire administration methods to check whether they are equivalent, since self-administration seems to be more coherent vis-à-vis the questions in the scale.^31^

One of the limitations of this study was the lack of a gold standard method to assess thirst intensity, a factor that might have yielded only moderate - nearly reasonable - agreement levels. The content validity index (CVI) was not computed as part of the analysis by the panel of experts. The lack of a CVI did not impair the comprehension of the TDS-BR. The recommendations made by the panel of experts were implemented to improve the clarity and relevance of each item in the scale. The panel of experts performed a qualitative assessment of the scale.

Although reproducibility analysis did not include the entire patient population, the partial population used in the study was enough to perform the analysis, since the magnitude and confidence interval supported a high intraclass correlation coefficient. Therefore, the size of the sample was enough to demonstrate the nearly perfect reproducibility of the scale.

The transcultural adaptation and psychometric validation of the TDS into the Brazilian context allowed the production of a concise and versatile instrument to assess thirst in patients on HD. Future studies may look into the administration of the scale in clinical trials and check for possible associations with interdialytic weight gain and food intake.

A positive correlation and moderate agreement were observed between the TDS-BR and the VAS, as seen in previous TDS validation studies.^16^^,^ 18 ^-^ 20 Content validity finds support in the literature and in the positive correlations and agreement established with the VAS.
